# Metabolomics and Lipidomics Study Unveils the Impact of Tauroursodeoxycholic Acid on Hyperlipidemic Mice

**DOI:** 10.3390/molecules28176352

**Published:** 2023-08-30

**Authors:** Na Cui, Wensen Zhang, Fazhi Su, Zhihong Zhang, Weijie Qiao, Yanping Sun, Bingyou Yang, Haixue Kuang, Qiuhong Wang

**Affiliations:** 1 Key Laboratory of Basic and Application Research of Beiyao, Ministry of Education, Heilongjiang University of Chinese Medicine, No. 24, Heping Road, Xiangfang District, Harbin 150040, China; 19866800702@163.com (N.C.); zhang1033362077@163.com (W.Z.); sfz18406564303@163.com (F.S.); zhihongzhang_555@126.com (Z.Z.); 18846836775@163.com (W.Q.); sunyanping_1@163.com (Y.S.); ybywater@hljucm.net (B.Y.); 2 Guangdong Engineering Technology Research Center for Standardized Processing of Chinese Materia Medica, School of Chinese Materia Medica, Guangdong Pharmaceutical University, No. 280, Waihuan East Road, Guangzhou 510006, China

**Keywords:** TUDCA, hyperlipidemia, metabolomics, glycerophospholipid metabolism, bile acids

## Abstract

Bear bile powder is an essential, traditional and valuable Chinese herbal medicine that clears heat, calms the liver, and improves eyesight. Early studies have shown that bear bile powder has lipid-lowering activity, but due to the scarcity of natural bear bile powder resources, it has yet to be used on a large scale. Researchers have found that tauroursodeoxycholic acid (TUDCA) is the primary characteristic bioactive substance of bear bile powder. This study aimed to investigate the therapeutic effect of TUDCA on high-fat diet (HFD)-induced hyperlipidemia. A hyperlipidemia model was established by feeding mice high-fat chow, following the intervention of different concentrations of TUDCA (25/50/100 mg/kg) orally, the hallmark biochemical indexes (total cholesterol (TC), total triglyceride (TG), high-density lipoprotein cholesterol (HDL-C), and low-density lipoprotein cholesterol (LDL-C)), histopathological examination (hematoxylin-eosin (HE) staining and oil red O (ORO) staining), and metabolomic analysis of serum and liver. The results showed that TUDCA could downregulate total TC, TG, LDL-C, upregulate HDL-C, reduce fat deposition in hepatocytes, reverse hepatocyte steatosis, and exhibit prominent lipid-lowering activity. In addition, it may play a therapeutic role by regulating glycerophospholipid metabolism.

## 1. Introduction

Dyslipidemia is one of the leading causes of metabolic syndrome, usually manifested as a decrease in high-density lipoprotein cholesterol (HDL-C) and an increase in total cholesterol (TC), total triglyceride (TG), and low-density lipoprotein cholesterol (LDL-C). Hyperlipidemia is also associated with fatty liver, obesity, and atherosclerosis [1,2] and affects many metabolic pathways, such as glucose metabolism, lipid metabolism, and bile acid metabolism. Hyperlipidemia has no clear clinical symptoms in its early stages, and its damage is insidious, progressive, and systemic. Hyperlipidemia-induced valve inflammation may be an essential initial process in aortic valve disease. The accumulation of oxidized LDL-C as the primary lipoprotein in the aortic valve leads to early aortic valve disease [3].

Lipids as a source of energy start with dietary fat intake, and then bile acids break down the fat and are absorbed by the intestinal lumen. In the intestinal cells, free-fatty acids combine with glycerol molecules to form TG, which accelerates the development of hyperlipidemia when chronic excess lipid intake and unhealthy lifestyle habits occur [4]. Excessive lipid intake may affect lipid transport in the hepatic-intestinal axis, and abnormal lipid metabolism in the intestine leads to excess lipid flow into the portal vein via the hepatic-intestinal circulation, resulting in lipid accumulation in the liver [5]. Lipid quadruple is a standard marker for the diagnosis of hyperlipidemia. Recent reports show that some institutions use a range of intelligent NIR luminol to diagnose hyperlipidemia. As a result, the design of innovative aggregates facilitates rapid and accurate detection of hyperlipidemia [6]. While the therapeutic effects of drugs are clearly defined, the safety of their administration has been of great concern. Statins are first-line drugs for the treatment of hyperlipidemia that help to reduce LDL-C and TG levels and increase HDL-C levels in patients with familial and severe hypercholesterolemia. Yet, they may be accompanied by adverse effects such as headache, myalgia, and hepatotoxicity. Patients may experience elevated alanine aminotransferase (ALT)/aspartate aminotransferase (AST) levels, increasing the risk of new-onset diabetes in adults [7]. Fenofibrate is also a commonly used clinically effective lipid-lowering drug with significant cholesterol and triglyceride-lowering effects and the ability to reduce residual cardiovascular risk, which is superior to statins alone [8,9]. Therefore, discovering potentially hypolipidemic active substances is an essential clinical objective.

Bile acids play an essential role as regulatory molecules in numerous biological processes. Combining taurine with bile acids helps to promote the breakdown of lipids and bile acids for fat and weight loss. TUDCA has been the focus of interest in recent years and is the primary bile acid in bear bile, a combination of taurine and ursodeoxycholic acid (UDCA). Studies have reported that TUDCA can reduce hepatic steatosis [10]. It is an endogenous chemical chaperone that protects cells from endoplasmic reticulum stress and treats NAFLD (nonalcoholic fatty liver disease) [11,12]. Slowing the progression of HFD (high-fat diet)-induced NAFLD by improving intestinal inflammation and barrier function reduces intestinal fat transport and modulates intestinal flora composition [13]. TUDCA penetrates the blood-brain barrier with low toxicity, and the US FDA has approved its precursor UDCA as a therapeutic agent for cholestatic liver disease [14]. Earlier studies had shown a linear and dose-dependent decrease in cholesterol saturation when duodenal bile was assayed after TUDCA use in gallstone patients [15,16], speculating that TUDCA may have a cholesterol-degrading effect. TUDCA also reduced intracellular cholesterol levels in chondrocytes and increased cell membrane fluidity [17]. This paper aims to explore the regulatory effect of TUDCA on hyperlipidemia mice through serum and liver metabolomics, in order to clarify the hypolipidemic activity and potential mechanism of TUDCA.

## 2. Results

### 2.1. Changes in Body Weight and Liver Index in Mice

**Figure 1:**
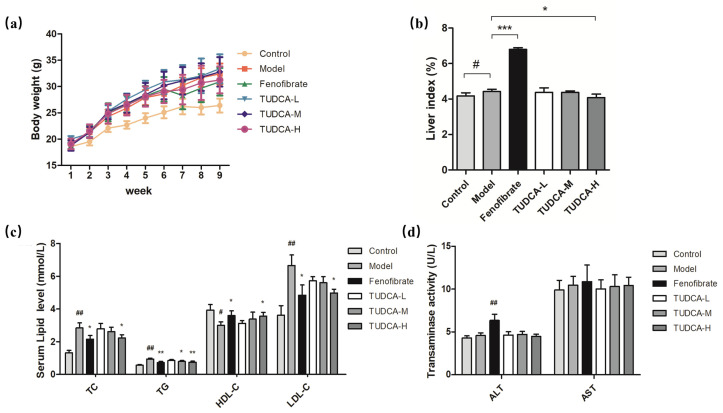
Changes in body weight and liver index in mice. (**a**) Body weight, (**b**) liver index, (**c**) levels of TC, TG, HDL-C, and LDL-C in serum, (**d**) ALT and AST vitality. Each value represents the mean ± SD. The error bar represents the SD of the data (*n* = 6). ## *p* < 0.01 or # *p* < 0.05 compared with the control group; *** *p* < 0.001, ** *p* < 0.01 or * *p* < 0.05 compared with the model group.

Compared with the control group, the hyperlipidemia model group’s body weight, liver index (*p* < 0.05), abdominal fat, and epididymal fat accumulation significantly increased. Compared with the hyperlipidemia model group, the body weight of each administration group did not improve considerably at the end of the experiment, the liver index of the fenofibrate group increased significantly (*p* < 0.001), and the TUDCA-H group decreased significantly (*p* < 0.05) (Figure 1a,b).

### 2.2. Physiological and Biochemical Indexes of Mice

To assess the effect of TUDCA on serum lipid levels and transaminases, we tested serum levels of TC, TG, HDL-C, LDL-C, and the viability of ALT and AST, respectively. Compared with the control group, the serum levels of TC, TG, and HDL-C in the model mice showed different degrees of increase (*p* < 0.05, *p* < 0.01) and LDL-C decreased (*p* < 0.01), whereas the vitality of ALT and AST did not change significantly. Compared with the model group, both fenofibrate and TUDCA-H showed a tendency to lower lipid levels (*p* < 0.05, *p* < 0.01), and TUDCA-M significantly decreased TG (*p* < 0.05). In the fenofibrate group, ALT activity increased (*p* < 0.01), which may be reflected in the side effects of drugs and may be related to the increase in the liver index (Figure 1c,d, Table S2).

### 2.3. Effect of TUDCA on Liver Histopathology in Mice

**Figure 2:**
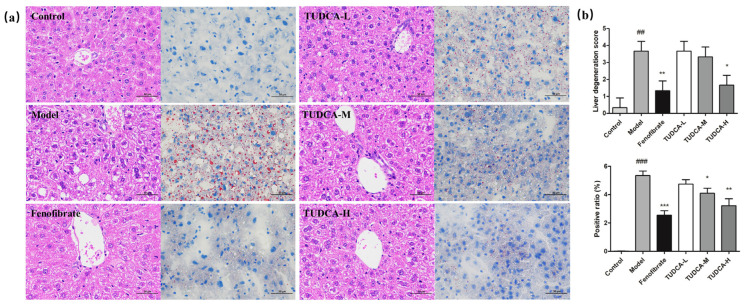
Observation of liver pathology in mice. (**a**) HE staining (400×) and ORO (400×) staining. (**b**) Liver pathology score and liver fat positivity rate. ## *p* < 0.01 or ### *p* < 0.001 compared with the control group; *** *p* < 0.001, ** *p* < 0.01 or * *p* < 0.05 compared with the model group.

Compared with the control group, the hepatocytes of the model group showed severe granular degeneration, cytoplasmic laxity, more vacuole distribution visible in the visual field, and more local hepatocyte steatosis as seen by large red positive ratios and reflected pathology scores and positive ratios (*p* < 0.01). Compared with the model group, TUDCA can slow down the steatosis of liver cells, make neutral fat deposition, and reduce the distribution. TUDCA-H plays a more significant role (*p* < 0.05, *p* < 0.01) (Figure 2a,b).

### 2.4. Multivariate Data Analysis of Serum Metabolomics and Lipidomics

**Figure 3:**
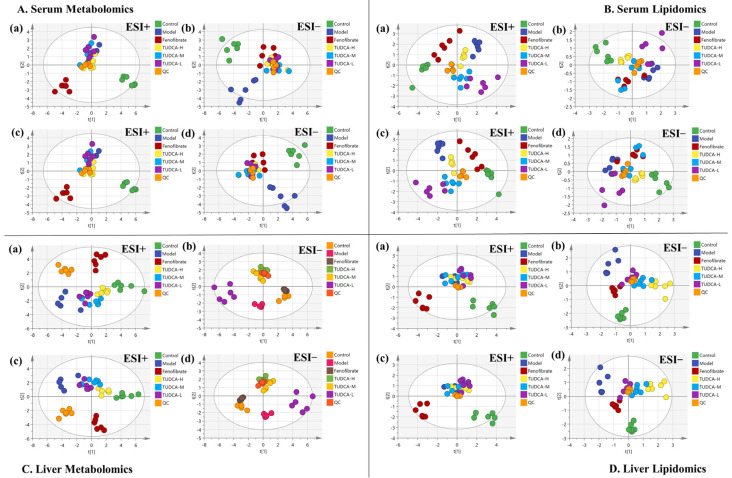
Effects of TUDCA on hyperlipidemic mice based on metabolic profile analysis. (**A**) Serum metabolomics, (**B**) serum lipidomics, (**C**) liver metabolomics, (**D**) liver lipidomics analysis, (**a**,**b**) PCA score plot in ESI positive and negative ion mode, (**c**,**d**) PLS−DA score plot in ESI positive and negative ion mode.

Using UPLC-Orbitrap/MS, a total ion characteristic diagram was generated based on MSI full scan data. The chemometric analysis of the dataset was performed using Xcalibur 4.3, Progenesis QI software V2.0, and Simca 14.1. To maximize the collected metabolism and lipidomics information and the fingerprint of the hyperlipidemia model, serum samples were obtained under the positive and negative modes of HESI. First, we performed unsupervised principal component analysis (PCA) on these data to observe the effect of TUDCA on the metabolic profile of the hyperlipidemia model. From the PLS-DA plot analysis, the different groups between the components were more prominent (Figure 3). As can be seen from Figure 3, there was a significant difference between the control and model groups, indicating that the whole body metabolic profile changed after 4 weeks. After administration of TUDCA, the hyperlipidemic mice in both positive and negative ion modes showed different degrees of improvement, with TUDCA-H probably showing the most pronounced effect.

### 2.5. The Endogenous Metabolites Identification and Correlation Analysis

Endogenous metabolites from PLS-DA analysis were obtained according to the VIP > 1 and *p* < 0.05 limits. Combined with the results of PCA, changes in metabolic status or metabolites were identified, which could be critical endogenous metabolites for metabolic pathways. A total of 15 endogenous metabolites (Table S3) in serum and 15 endogenous metabolites (Table S5) in the liver were identified, these markers were closely associated with hyperlipidemia models and TUDCA for metabolomics analysis. A total of 14 endogenous metabolites (Table S4) in serum and 11 endogenous metabolites (Table S6) were identified in the liver, these markers were closely associated with hyperlipidemia models and TUDCA for lipidomics analysis.

**Figure 4:**
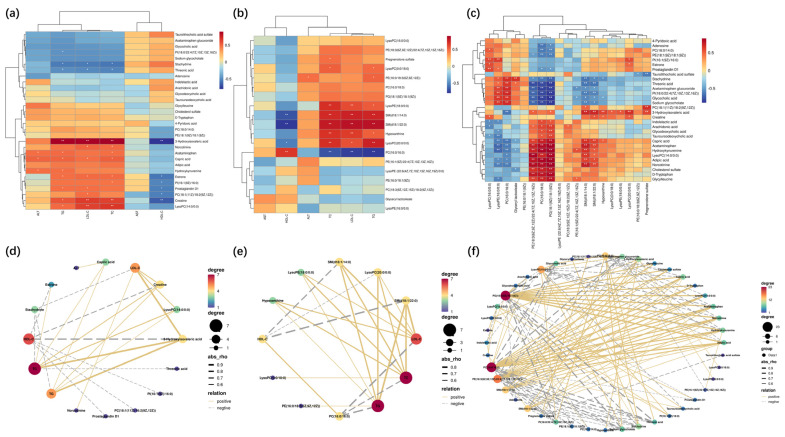
Spearman correlation analysis. (**a**,**d**) Serum biochemical indices with serum and liver endogenous metabolites. (**b**,**e**) Serum biochemical indices with serum and liver endogenous lipid metabolites. (**c**,**f**). Serum and liver endogenous metabolites versus serum and liver endogenous lipid metabolites. In Spearman correlation analysis (**a**–**c**), warm color represents positive correlation and cold color represents negative correlation; * *p* < 0.05, ** *p* < 0.01 indicate the significance of serum indices correlated with metabolites. In the correlation network diagrams (**d**–**f**), solid lines represent positive correlations, dashed lines represent positive correlations, and the size or color of the dots represent the number of correlated objects.

We conducted Spearman correlation tests to explore the relationship between metabolites and physiologic characteristics. A total of 30 serum and liver metabolites were correlated with physiological characteristics associated with hyperlipidemia (e.g., HDL-C, LDL-C, TC, TG, etc., Figure 4a,d). Out of these metabolites, 11 showed significant positive correlations with LDL-C, TC, and TG, whereas 6 metabolites showed significant negative correlations with HDL-C. Creatine and 3-hydroxyisovaleric acid showed the most significant correlations with lipid indices. The relationships between metabolites and lipid indices (HDL−C, LDL−C, TC, TG) were stronger than those of liver indices (AST and ALT). A total of 19 different serum and hepatic lipid metabolites were correlated with physiological features associated with hyperlipidemia (e.g., HDL−C, LDL−C, TC, TG, etc., Figure 4b,e). Eight different metabolites showed significant positive correlations with LDL−C, TC, and TG, whereas three metabolites showed negative correlations with HDL−C. SM (d18:1/14:0) and SM (d18:1/22:0) showed significant correlations with lipid indices. Finally, metabolites and lipid metabolites were correlated (Figure 4c,f).

### 2.6. The Pathway Enrichment Analysis and Metabolic Network

**Figure 5:**
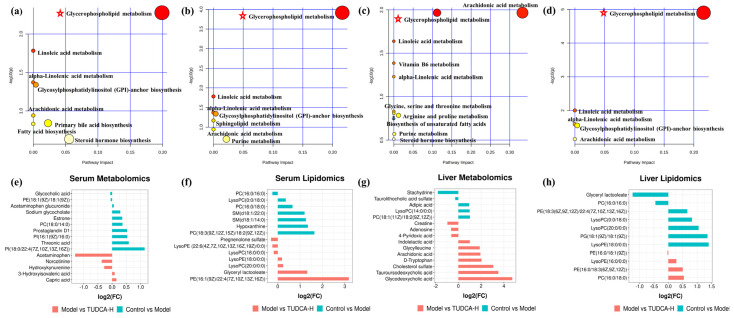
The function enrichment analysis of the TUDCA−H affected by the hyperlipidemia model. (**a**,**e**) Serum metabolomics, (**b**,**f**) serum lipidomics, (**c**,**g**) liver metabolomics, (**d**,**h**) liver lipidomics. ✩, Represents important metabolic pathways.

**Figure 6:**
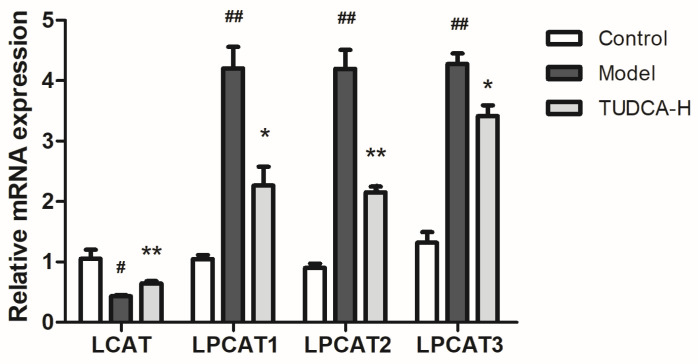
Effect of TUDCA−H on LCAT, LPCAT1, LPCAT2, LPCAT3 mRNA expression levels in HFD−induced mouse liver tissue (*n* = 3, means ± SD). Calculation of the relative expression of mRNA using the 2^−ΔΔC(t)^ algorithm. One−way ANOVA was used. # *p* < 0.05 or ## *p* < 0.01 compared with the control group; * *p* < 0.05 or ** *p* < 0.01 compared with the model group.

The metabolic pathways associated with these differential metabolites were analyzed through MetaboAnalyst, searched and annotated by the KEGG database of endogenous metabolites, and correlation analysis of related pathways and targets was carried out. The metabolomics and lipidomics analysis of serum and liver revealed the most significant impact on glycerophospholipid metabolism, indicating that glycerophospholipid metabolism is closely associated with hyperlipidemia and TUDCA, and is one of the critical metabolic pathways (Figure 5). RT−qPCR verified the mRNA expression levels of LCAT (lecithin−cholesterol acyltransferase), LPCAT1 (lysophosphatidylcholine acyltransferase 1), LPCAT2, and LPCAT3, which are the key enzymes of the pathway, and the results showed that compared with the model group, TUDCA−H was able to significantly upregulate the mRNA expression levels of LCAT, and downregulate the mRNA expression levels of LPCAT1, LPCAT2, and LPCAT3 (Figure 6).

## 3. Discussion

Fenofibrate can treat hypercholesterolemia and hypertriglyceridemia [18]. In this study, we have selected fenofibrate as a positive control group. Weight gain was insignificant, lipid levels improved most significantly during administration, and the elevated liver index and ALT were probably due to high doses. The safety of medication use is becoming increasingly important; therefore, it is essential to identify safe, non-toxic, and effective lipid−lowering agents. As bile acids can promote the digestion and absorption of lipids and inhibit the precipitation of cholesterol in the bile, preventing the formation of gallstones [19], TUDCA, bear bile’s primary bile acid component, was chosen to investigate the lipid−lowering effects in this study.

Mammals’ most abundant bile acids (BAs) include primary and secondary bile acids. BAs are synthesized in hepatocytes via cytochrome P450 (CYP450)−mediated cholesterol oxidation [20,21]. In hepatocytes, most BAs are bound to glycine or taurine by the action of bile acid CoA synthase (BACS) and amino acid N−acyltransferase (BAAT), and then secreted into the bile via the bile salt export pump (BSEP). Bile acids have direct or indirect antimicrobial effects and they can modulate the microbiota composition, as shown by the size and design of the bile acid pool [22]. The discovery of TUDCA and TCDCA in the metabolic profile of bile acids reverses the activation of FXR signaling by CDCA through reducing the FXR fraction in the nucleus. It may hold promise as a viable therapy for treating HFD-induced obesity or hypercholesterolemia [23,24]. Recent studies have shown that targeting the hepatic−intestinal axis and bile acid analogs are potential therapeutic approaches for treating NAFLD. TUDCA attenuated hepatic steatosis in NAFLD mice and attenuated HFD−induced NAFLD progression in mice by improving intestinal inflammation and barrier, thereby reducing intestinal fat transport and modulating intestinal microbiota composition [25]. The ability of TUDCA to reduce the mitochondrial levels of long−chain acyl−CoA dehydrogenase (LCDA), which in turn reduces the beta−oxidation of long-chain fatty acids, and to regulate the levels of sterol regulatory element binding protein (SREBP-1) were also identified in studies on the neuroprotection of TUDCA [26]. TUDCA can reverse the metabolic disturbances induced by HFD feeding and contribute to its hepatoprotective effects by regulating osmolality and cellular signaling to control glucose, lipids, and metabolites involved in methionine and homocysteine metabolism, thereby reducing oxidative stress and endoplasmic reticulum stress [27]. The present study also demonstrated the normalization of lipid levels and a reduction in hepatic steatosis after oral administration of TUDCA, which is consistent with the literature.

Metabolomics and lipidomics provide comprehensive information on endogenous molecules in the body, which is crucial for early disease diagnosis [28]. Biomarkers are unbiased differential indicators for classifying disease progression and drug efficacy [29,30]. Serum biomarkers, such as TC and TG, use glucose tolerance to diagnose lipid disorders. Some emerging biomarkers can provide valuable information, such as APO1, FFA, lipid transport proteins, and growth hormone-releasing hormone [31]. In this study, different metabolites in serum, liver, serum lipids, and liver lipids were detected by UPLC-MS, and metabolic networks were analyzed. The results showed that glycerophospholipid metabolism was the most critical in serum, liver metabolism, and lipid metabolism in hyperlipidemia with TUDCA intervention. The literature also reported the relationship between potential biomarkers of creatine. A study published in the Dutch Medical Journal suggests that taking a high dose of creatine can increase triglyceride levels in the body, thereby increasing the risk of hyperlipidemia. In addition, the study found that compared to the placebo group, people who took a high dose of creatine for a long time had elevated levels of total cholesterol and low-density lipoprotein cholesterol in their serum [32]. Adding a diet containing linolenic acid to a high-fat diet in mice can significantly increase their blood prostaglandin D1 levels and lower their cholesterol and triglyceride levels. In addition, some human studies have shown that linolenic acid and prostaglandin D1 can reduce fat blood levels by regulating the metabolism of adipocytes, thereby reducing the risk of developing hyperlipidemia [33]. A high-fat diet can significantly increase the content of SM (d18:1/22:0) in the liver and blood of mice, which also leads to an increase in lipid levels in the blood of mice and an increase in liver fat accumulation. These results indicate that the expansion of SM (d18:1/22:0) may be involved in lipid metabolism abnormalities and fatty liver induced by a high-fat diet [34]. A high-fat diet can significantly increase the content of SM (d18:1/14:0) in the liver and blood of mice, as well as increase the levels of triglycerides and cholesterol in the blood of mice [35] and glucose metabolism and lipid metabolism.

We examined critical enzymes in the metabolism of glycerophospholipids, such as LCAT (lecithin-cholesterol acyltransferase) and LPCAT (lysophosphatidylcholine acyltransferases), which esterify free cholesterol to cholesteryl esters, a lipid that can bind to other lipids to form cholesteryl ester particles, making them more easily translocated to the liver and lowering their concentration in the blood. This also facilitates the binding of phosphatidylcholine and cholesterol, resulting in the formation of phosphatidylcholine/cholesterol complexes. These complexes are further metabolized in the liver. LCAT plays a central role in intravascular HDL metabolism [36]. Several studies have suggested that LCAT expression and activity may be downregulated in specific hyperlipidemia symptoms [37]. For example, one study found that LCAT expression levels were downregulated in some patients with hereditary hypercholesterolemia, possibly due to abnormal LCAT function due to genetic mutations [38]. In addition, some studies have found that LCAT expression and activity are downregulated in some cases of obesity and metabolic syndrome, possibly due to chronic low-grade inflammation, insulin resistance, and other factors [39,40]. The LPCAT family refers to the phosphatidylcholine (PC) transferase family. It consists of four members: LPCAT1, LPCAT2, LPCAT3, and LPCAT4. This type of enzyme plays a crucial role in the re-acylation reaction in phospholipid remodeling. It can catalyze the esterification reaction between lysophospholipid and phosphatidylcholine to generate PC. Changing the length and type of fatty acids at specific sites in phospholipids enables the conversion of nascent phospholipids to mature phospholipids, which is a critical step in phospholipid metabolism [41,42]. In addition, the LPCAT family is involved in physiological and pathological processes, such as insulin signaling pathways, brain neuron formation and maintenance, hepatic lipid metabolism, and obesity [43].

**Figure 7:**
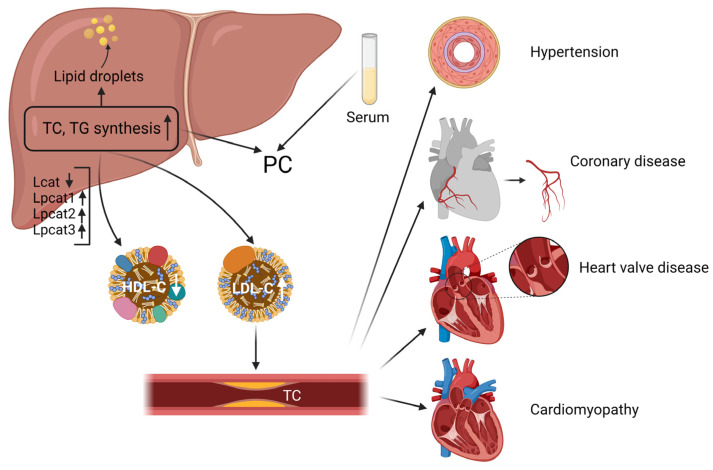
The liver serves as the central organ of hyperlipidemia.

Among the LPCAT family members, LPCAT1 preferentially integrates saturated fatty acids into PCs, whereas LPCAT3 preferentially integrates PUFA into PCs. LPCAT3 is a phospholipid (PL) remodeling enzyme that produces polyunsaturated PLs, a significant determinant of membrane PC content in the liver. It is also a potential target for treating metabolic disorders such as hyperlipidemia and atherosclerosis [41]. Reduced LPCAT3 expression has also been shown to enhance endoplasmic reticulum stress and hepatocyte injury [44]. LPCAT3 is significantly inhibited in human NASH livers, and LPCAT3-deficiency in mouse livers promotes both spontaneous and dietary-induced NASH/HCC. LPCAT3 overexpression ameliorates hepatic inflammation and fibrosis [45,46]. In conclusion, the LCAT and LPCAT families play essential roles in maintaining normal lipid metabolism and cell membrane structure and function. However, the targeting and regulation of this crucial enzyme by TUDCA may also be a critical lipid-lowering mechanism by TUDCA—supplementary TUDCA-related research literature (Figure 7).

## 4. Materials and Methods

### 4.1. Animals, Diets, and Ethics Statement

SPF-grade male C57BL/6 mice (7 ± 1 weeks old, purchased from Guangdong Medical Laboratory Animal Center, China, License No. SCXK (Guangdong) 2022-0002) were housed under standard conditions (12/12 h light/dark alternation, temperature of 22 ± 2 °C, relative humidity of 50 ± 10%). After 1 week of adaptive feeding and drinking, the group randomly divided mice into 6 groups (Control, Model, Fenofibrate, TUDCA-L, TUDCA-M, and TUDCA-H) (*n* = 8). We provided the control group with a control diet (purchased from Guangdong Medical Laboratory Animal Center, license number: Guangdong Feeding Certificate (2019) 05073) and provided other groups with high-fat diet (purchased from Jiangsu Synergy Pharmaceutical and Biological Engineering Co., Ltd., batch number: XTHF45, composition: 22.5% crude protein, 24.2% crude fat, 3.2% crude fiber, 5.6% crude ash, 1.2% calcium, and 0.8% total phosphorus). The control group of mice was fed a diet formulation of 18.5% protein, 4.6% fat, 58.9% carbohydrate, 3.2% crude fiber, 6.8% ash, 1.28% calcium, and 0.92 phosphorus. We conducted our experiments in accordance with national and European Union guidelines for the handling and use of laboratory animals, as well as studies and protocols approved by the Animal Ethics Committee of the Guangdong Provincial Medical Laboratory Animal Center (approval number: C202211-1). The minimal possible number of animals was sacrificed, while all efforts were made to reduce their suffering.

### 4.2. Chemicals and Reagents

We purchased TUDCA from APExBIO (batch number: C3233). AST, ALT, TC, TG, LDL-C, and HDL-C kits were from Nanjing Jiancheng Institute of Biological Engineering, Nanjing, China (batch number: 20220830). HE and ORO are all from Servicebio, Wuhan, China (batch number: G1003, G1015). Mass spectrometry grade formic acid and acetonitrile were from Thermo Fisher (batch number: 205178, 205187). Primer synthesis was commissioned to Shanghai Bioengineering Co., Shanghai, China.

### 4.3. Establishment of the Hyperlipidemia Model

**Figure 8:**
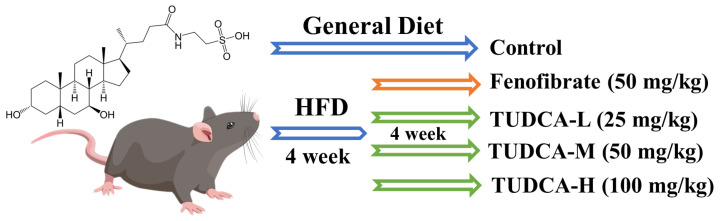
Establishment of hyperlipidemia model and drug administration intervention process.

Except for the control group, all groups had a high-fat diet. Blood was collected from mice’s tail tips 4 weeks later, and serum lipid levels were measured (TC, TG, HDL-C, LDL-C). All groups were given equal volumes of fenofibrate (50 mg/kg), TUDCA-L (25 mg/kg), TUDCA-M (50 mg/kg), and TUDCA-H (100 mg/kg) orally until the 8th week and saline was given to the control group and model group orally on a daily basis. Then, body weight and food intake of each animal were measured weekly during this period (Figure 8). At the end of the final treatment, mice were fasted without water for 12 h and then injected intraperitoneally with 0.3% sodium pentobarbital at an anesthetic dosage of 50 mg/kg. After fully anesthetizing the mice, we collected all blood by orbital sinus blood sampling. The target tissues were collected, and the animal carcasses were disposed of properly.

### 4.4. Detection of Serum Biochemical Indexes

ELISA measured mouse serum TC, TG, LDL-C, HDL-C, AST, and ALT levels.

### 4.5. Histopathological Examination

Liver tissue was analyzed using hematoxylin-eosin (HE) and oil red O (ORO) staining. A portion of the mouse liver was fixed in 4% paraformaldehyde solution, paraffin-embedded, and stained with HE. The other part was freeze-embedded for frozen sections and stained with ORO. To capture images of the samples, the areas were photographed with a microscope (Nikon (Eclipse Ci-L)) (400× microscopic observation).

### 4.6. Preparation of Untargeted Serum Metabolomics and Lipidomics Samples

The serum or liver tissue was added to acetonitrile at a volume ratio of 1:3 and centrifuged at 13,000 r/min for 10 min at 4 °C. The evaporated supernatant and the sample were re-solubilized with 50% acetonitrile. After high-speed centrifugation, the supernatant was taken for detection, preparation, and quality control (QC) by mixing 10 μL of each sample. Then, we inserted a QC sample in every 7 samples to check the stability and reproducibility of the system for metabolomics analysis.

First, we added the serum or liver tissue (50 μL) to iced methanol (200 μL) at a volume ratio of 1:4 and vortexed for 60 s. Then, we used the iced MTBE (1000 μL) and vortexed for 60 s. Finally, we added the deionized water (200 μL) and vortexed for 300 s and centrifuged at 4 °C for 10 min at 13,500 r/min. The upper lipid extraction fraction was collected and blow-dried under nitrogen at 40 °C. Then, a buffer containing internal standards (hexadecanoic acid-d31, d5 TG (16:0/18:0/16:0), d31 PC (16:0/18:1), cholesterol-d7, Cer (d18:1/16:0), and SM (d18:1/17:0), each with 500 ng) were passed through 200 μL of isopropanol/acetonitrile (1: 1, *v*/*v*) to re-solubilize the samples for lipidomics analysis.

### 4.7. UPLC-Orbitrap/MS Analysis

Samples were detected using an ultra-high performance liquid phase (Dionex Ultimate 3000, Thermo, Waltham, MA, USA) tandem electrostatic field orbitrap high-resolution mass spectrometer (Thermo Orbitrap Fusion, Mundelein, IL, USA). Serum samples were subjected to the following: Gradient elution on a Waters ACQUITY UPLC HSS T3 (1.8 μm, 2.1 × 100 mm) column at a 0.4 mL/min flow rate; ion source type (H-ESI) in negative ion mode at 2.8 kV; ion transfer tube temp: 300 °C; vaporizer temp: 320 °C; sheath gas: 20 Arb; auxiliary gas: 6 Arb; mass range: 100–1000 m/z; fragment energies of 15%, 25%, and 35% with a resolution of 15,000; dynamic rendition time of 6 s. The stability of the analysis was monitored continuously by systematically analyzing QC samples every 10samples. The mobile phases comprised water, 0.1% formic acid (phase A), and acetonitrile (phase B). Elute phase B gradients for metabolomics analysis were as follows: 2% (0–1 min), 2% to 35% (1–4 min), 35% to 100% (4–13 min), 100% to 100% (13–15.5 min), 100% to 2% (15.5–19 min). The mobile phases consisted of acetonitrile: water (3:2, 10 mM ammonium formate, 0.1% formic acid) (phase A) and isopropanol: acetonitrile (9:1, 10 mM ammonium formate, 0.1% formic acid) (phase B). Elute phase B gradients for lipidomics analysis were as follows: 20% (0 min), 20% to 65% (0–4 min), 65% to 80% (4–8 min), 80% to 95% (8–11 min), 95% to 95% (11–12 min), 95% to 20% (12–13 min), 20% to 20% (13–15 min). After obtaining the dataset of MS scans, this dataset was integrated and analyzed with *p* < 0.05, VIP > 1 as the screening criteria. Then, we screened the key differential metabolites through HMDB (https://hmdb.ca/ (accessed on 5 June 2023)) and LIPID MAPS (https://www.lipidmaps.org/) websites.

### 4.8. Key Targets for Quantitative Real-Time Polymerase Chain Reaction (RT-qPCR) and Western Blotting Validation

Based on the results of bioinformatics analysis, the expression levels of the most critical related genes in the pathway were verified by RT-qPCR and Western blotting. Total liver tissue RNA was extracted and reverse transcribed using SteadyPure Universal RNA Extraction Kit and Reverse Transcription Kit (Hunan Acres Bioengineering Co., Ltd., Changsha, China). We set up the RT-qPCR amplification procedure as follows: After completing the amplification reaction, the melting curves of PCR products were plotted (95 °C for 15 s, 60 °C for 15 s, and 95 °C for 15 s). In addition, 18srRNA was used as an internal reference gene, and the relative expression was calculated as 2^−ΔΔC(t)^ method. Primer sequences are shown in Table S1.

### 4.9. Statistical Analysis

Details and assessments of histopathological scores and biochemical index tests were analyzed by GraphPad Prism 5 (GraphPad Software 5.01, San Diego, CA, USA), expressed as mean ± standard deviation (SD), and used *p* < 0.05 and *p* < 0.01 to evaluate significance. Serum and liver metabolic data were collected and analyzed by Thermo Xcalibur 4.3 software, Simca, and Progressive QI software (Waters, Milford, MA, USA). Endogenous metabolites were identified by principal component analysis (PCA), dimension reduction, and partial least squares discriminant analysis (PLS-DA). We screened the objective values for components that significantly affected grouping (*p* < 0.05) and found key metabolic pathways using MetaboAnalyst (www.metaboanalyst.ca (accessed on 10 June 2023)).

## 5. Conclusions

In this study, it was reported for the first time that different concentrations of TUDCA improved hyperlipidemia in male mice to varying degrees, and TUDCA-H could significantly regulate TC, TG, HDL-C, and LDL-C. We obtained the metabolic pathways and targets for treating hyperlipidemia with TUDCA. Among them, glycerophospholipid metabolism is the most critical pathway. TUDCA plays an important therapeutic role and can be used as a potential medicinal drug for hyperlipidemia.

## Data Availability

The data supporting the findings of the present study are not publicly available. The data are available only upon reasonable request and with permission from the authors.

## Supplementary Materials

The following supporting information can be downloaded at: https://www.mdpi.com/article/10.3390/molecules28176352/s1.

## Abbreviations

HFD, high-fat diet; TUDCA, Tauroursodeoxycholic acid; TC, total cholesterol; TG, triglycerides; HDL-C, high-density lipoprotein cholesterol; LDL-C, low-density lipoprotein cholesterol; ALT, Alanine aminotransferase; AST, Aspartate aminotransferase; HE, Hematoxylin and Eosin; ORO, oil red O; PCA, Principal Component Analysis; PLS-DA, Partial Least Squares Discrimination Analysis; KEGG, Kyoto Encyclopedia of Genes and Genomes; VIP, Variable importance in the projection; QC, quality control.

## References

[1] Libby P., Buring J.E., Badimon L., Hansson G.K., Deanfield J., Bittencourt M.S., Tokgözoğlu L., Lewis E.F. (2019). Atherosclerosis. Nat. Rev. Dis. Primers.

[2] Targher G., Byrne C.D., Lonardo A., Zoppini G., Barbui C. (2016). Non-alcoholic fatty liver disease and risk of incident cardiovascular disease: A meta-analysis. J. Hepatol..

[3] Lee S.H., Kim N., Kim M., Woo S.H., Han I., Park J., Kim K., Park K.S., Kim K., Shim D. (2022). Single-cell transcriptomics reveal cellular diversity of aortic valve and the immunomodulation by PPARγ during hyperlipidemia. Nat. Commun..

[4] Djordjevic D.B., Zdravkovic M., Nagorni A., Manolis A., Tsioufis C., Lovic D. (2018). A Critical Approach of Guideline Therapeutic Recommendations for NAFLD. Curr. Vasc. Pharmacol..

[5] Hannah W.N., Harrison S.A. (2016). Noninvasive imaging methods to determine severity of nonalcoholic fatty liver disease and nonalcoholic steatohepatitis. Hepatology.

[6] Sun F., Zhao W., Shen H., Fan N., Zhang J., Liu Q., Xu C., Luo J., Zhao M., Chen Y. (2022). Design of Smart Aggregates: Toward Rapid Clinical Diagnosis of Hyperlipidemia in Human Blood. Adv. Mater..

[7] Stewart J., McCallin T., Martinez J., Chacko S., Yusuf S. (2020). Hyperlipidemia. Pediatr. Rev..

[8] Jin L., Hua H., Ji Y., Jia Z., Peng M., Huang S. (2023). Anti-inflammatory role of fenofibrate in treating diseases. Biomol. Biomed..

[9] Park M.S., Youn J.C., Kim E.J., Han K.H., Lee S.H., Kim S.H., Kim B.J., Kwon S.U., Ryu K.H. (2021). Efficacy and Safety of Fenofibrate-Statin Combination Therapy in Patients With Inadequately Controlled Triglyceride Levels Despite Previous Statin Monotherapy: A Multicenter, Randomized, Double-blind, Phase IV Study. Clin. Ther..

[10] Legry V., Van Rooyen D.M., Lambert B., Sempoux C., Poekes L., Español-Suñer R., Molendi-Coste O., Horsmans Y., Farrell G.C., Leclercq I.A. (2014). Endoplasmic reticulum stress does not contribute to steatohepatitis in obese and insulin-resistant high-fat-diet-fed foz/foz mice. Clin. Sci..

[11] Choi Y.J., Shin H.S., Choi H.S., Park J.W., Jo I., Oh E.S., Lee K.Y., Lee B.H., Johnson R.J., Kang D.H. (2014). Uric acid induces fat accumulation via generation of endoplasmic reticulum stress and SREBP-1c activation in hepatocytes. Lab. Investig. J. Tech. Methods Pathol..

[12] Itoh H., Muramatsu-Kato K., Ferdous U.J., Kohmura-Kobayashi Y., Kanayama N. (2017). Undernourishment in utero and hepatic steatosis in later life: A potential issue in Japanese people. Congenit. Anom..

[13] Wang W., Zhao J., Gui W., Sun D., Dai H., Xiao L., Chu H., Du F., Zhu Q., Schnabl B. (2018). Tauroursodeoxycholic acid inhibits intestinal inflammation and barrier disruption in mice with non-alcoholic fatty liver disease. Br. J. Pharmacol..

[14] Zangerolamo L., Vettorazzi J.F., Rosa L.R.O., Carneiro E.M., Barbosa H.C.L. (2021). The bile acid TUDCA and neurodegenerative disorders: An overview. Life Sci..

[15] Muraca M., Vilei M.T., Cianci V., Liu X.T. (1995). Effect of tauroursodeoxycholic acid (TUDCA) on biliary lipid composition. Ital. J. Gastroenterol..

[16] Lu Q., Jiang Z., Wang Q., Hu H., Zhao G. (2021). The effect of Tauroursodeoxycholic acid (TUDCA) and gut microbiota on murine gallbladder stone formation. Ann. Hepatol..

[17] Arai Y., Choi B., Kim B.J., Rim W., Park S., Park H., Ahn J., Lee S.H. (2019). Tauroursodeoxycholic acid (TUDCA) counters osteoarthritis by regulating intracellular cholesterol levels and membrane fluidity of degenerated chondrocytes. Biomater. Sci..

[18] Sidhu G., Tripp J. (2023). Fenofibrate. StatPearls.

[19] Kusaczuk M. (2019). Tauroursodeoxycholate-Bile Acid with Chaperoning Activity: Molecular and Cellular Effects and Therapeutic Perspectives. Cells.

[20] Jia W., Xie G., Jia W. (2018). Bile acid-microbiota crosstalk in gastrointestinal inflammation and carcinogenesis. Nat. Rev. Gastroenterol. Hepatol..

[21] Axelson M., Ellis E., Mörk B., Garmark K., Abrahamsson A., Björkhem I., Ericzon B.G., Einarsson C. (2000). Bile acid synthesis in cultured human hepatocytes: Support for an alternative biosynthetic pathway to cholic acid. Hepatology.

[22] Huang F., Zheng X., Ma X., Jiang R., Zhou W., Zhou S., Zhang Y., Lei S., Wang S., Kuang J. (2019). Theabrownin from Pu-erh tea attenuates hypercholesterolemia via modulation of gut microbiota and bile acid metabolism. Nat. Commun..

[23] Chiang J.Y.L. (2017). Bile acid metabolism and signaling in liver disease and therapy. Liver Res..

[24] Wang K., Liao M., Zhou N., Bao L., Ma K., Zheng Z., Wang Y., Liu C., Wang W., Wang J. (2019). Parabacteroides distasonis Alleviates Obesity and Metabolic Dysfunctions via Production of Succinate and Secondary Bile Acids. Cell Rep..

[25] Dos Reis Araujo T., Santiago D., Simões P., Guimarães F., Zoppi C.C., Carneiro E.M. (2022). The Taurine-Conjugated Bile Acid (TUDCA) Normalizes Insulin Secretion in Pancreatic β-Cells Exposed to Fatty Acids: The Role of Mitochondrial Metabolism. Adv. Exp. Med. Biol..

[26] Fernandes M.B., Costa M., Ribeiro M.F., Siquenique S., Sá Santos S., Martins J., Coelho A.V., Silva M.F.B., Rodrigues C.M.P., Solá S. (2020). Reprogramming of Lipid Metabolism as a New Driving Force Behind Tauroursodeoxycholic Acid-Induced Neural Stem Cell Proliferation. Front. Cell Dev. Biol..

[27] Sun R., Xu D., Wei Q., Zhang B., Aa J., Wang G., Xie Y. (2020). Silybin ameliorates hepatic lipid accumulation and modulates global metabolism in an NAFLD mouse model. Biomed. Pharmacother..

[28] Zhang A., Sun H., Wang X. (2012). Serum metabolomics as a novel diagnostic approach for disease: A systematic review. Anal. Bioanal. Chem..

[29] González-Domínguez R., García A., García-Barrera T., Barbas C., Gómez-Ariza J.L. (2014). Metabolomic profiling of serum in the progression of Alzheimer’s disease by capillary electrophoresis-mass spectrometry. Electrophoresis.

[30] Shah V.O., Townsend R.R., Feldman H.I., Pappan K.L., Kensicki E., Vander Jagt D.L. (2013). Plasma metabolomic profiles in different stages of CKD. Clin. J. Am. Soc. Nephrol. CJASN.

[31] Mato J.M., Alonso C., Noureddin M., Lu S.C. (2019). Biomarkers and subtypes of deranged lipid metabolism in non-alcoholic fatty liver disease. World J. Gastroenterol..

[32] Kreider R.B., Melton C., Rasmussen C.J., Greenwood M., Lancaster S., Cantler E.C., Milnor P., Almada A.L. (2003). Long-term creatine supplementation does not significantly affect clinical markers of health in athletes. Mol. Cell. Biochem..

[33] Serhan C.N., Clish C.B., Brannon J., Colgan S.P., Chiang N., Gronert K. (2000). Novel functional sets of lipid-derived mediators with antiinflammatory actions generated from omega-3 fatty acids via cyclooxygenase 2-nonsteroidal antiinflammatory drugs and transcellular processing. J. Exp. Med..

[34] Wang Z., Klipfell E., Bennett B.J., Koeth R., Levison B.S., Dugar B., Feldstein A.E., Britt E.B., Fu X., Chung Y.M. (2011). Gut flora metabolism of phosphatidylcholine promotes cardiovascular disease. Nature.

[35] Brügger B. (2014). Lipidomics: Analysis of the lipid composition of cells and subcellular organelles by electrospray ionization mass spectrometry. Annu. Rev. Biochem..

[36] Ossoli A., Strazzella A., Rottoli D., Zanchi C., Locatelli M., Zoja C., Simonelli S., Veglia F., Barbaras R., Tupin C. (2021). CER-001 ameliorates lipid profile and kidney disease in a mouse model of familial LCAT deficiency. Metab. Clin. Exp..

[37] Laurenzi T., Parravicini C., Palazzolo L., Guerrini U., Gianazza E., Calabresi L., Eberini I. (2021). rHDL modeling and the anchoring mechanism of LCAT activation. J. Lipid Res..

[38] Wu W., Hu Y., Zhang S., Liu D., Li Q., Lin Y., Liu Z. (2021). Untargeted metabolomic and lipid metabolism-related gene expression analyses of the effects and mechanism of aged Liupao tea treatment in HFD-induced obese mice. RSC Adv..

[39] Ashokkumar N., Vinothiya K. (2023). Protective Impact of Vanillic Acid on Lipid Profile and Lipid Metabolic Enzymes in Diabetic Hypertensive Rat Model Generated by A High-Fat Diet. Curr. Drug Discov. Technol..

[40] Zhu Y., Wei Y.L., Karras I., Cai P.J., Xiao Y.H., Jia C.L., Qian X.L., Zhu S.Y., Zheng L.J., Hu X. (2022). Modulation of the gut microbiota and lipidomic profiles by black chokeberry (Aronia melanocarpa L.) polyphenols via the glycerophospholipid metabolism signaling pathway. Front. Nutr..

[41] Wang B., Tontonoz P. (2019). Phospholipid Remodeling in Physiology and Disease. Annu. Rev. Physiol..

[42] Zhang Q., Yao D., Rao B., Jian L., Chen Y., Hu K., Xia Y., Li S., Shen Y., Qin A. (2021). The structural basis for the phospholipid remodeling by lysophosphatidylcholine acyltransferase 3. Nat. Commun..

[43] Law S.H., Chan M.L., Marathe G.K., Parveen F., Chen C.H., Ke L.Y. (2019). An Updated Review of Lysophosphatidylcholine Metabolism in Human Diseases. Int. J. Mol. Sci..

[44] Kawamura S., Matsushita Y., Kurosaki S., Tange M., Fujiwara N., Hayata Y., Hayakawa Y., Suzuki N., Hata M., Tsuboi M. (2022). Inhibiting SCAP/SREBP exacerbates liver injury and carcinogenesis in murine nonalcoholic steatohepatitis. J. Clin. Investig..

[45] Tian Y., Jellinek M.J., Mehta K., Seok S.M., Kuo S.H., Lu W., Shi R., Lee R., Lau G.W., Kemper J.K. (2023). Membrane phospholipid remodeling modulates nonalcoholic steatohepatitis progression by regulating mitochondrial homeostasis. Hepatology.

[46] Kakisaka K., Suzuki Y., Fujiwara Y., Suzuki A., Kanazawa J., Takikawa Y. (2019). Caspase-independent hepatocyte death: A result of the decrease of lysophosphatidylcholine acyltransferase 3 in non-alcoholic steatohepatitis. J. Gastroenterol. Hepatol..

