# Supplementary material for: Metabolomics and Lipidomics Study Unveils the Impact of Tauroursodeoxycholic Acid on Hyperlipidemic Mice
Source: Molecules. 2023 Aug 30;28(17):6352. doi: 10.3390/molecules28176352 (PMC10490038; doi:10.3390/molecules28176352)
Supplement: Supplementary file 1 [file molecules-28-06352-s001.zip › molecules-2580207-supplementary.pdf]

Table S1. The sequences of primers for LCAT, LPCAT1, LPCAT2, LPCAT3 and 18S used in this study.

Gene Name	Forward Primers (5'-3')	Reverse Primers (5'-3')
LCAT	CTGGCTCCTCAATGTGCTCTTC	AGAAGTCCTCTGTCTTACGGTAGC
LPCAT 1	AGAGTGGTGGTGGTGAGATTGAC	CTGGATGGTGGCTAAGGTCTGG
LPCAT 2	TGAGGACGGCTACATAACAGAGG	CTGGGTGCTTTAGGGCGAAAC
LPCAT3	AAGCAGGTCAGCAGTCTAATTCG	GGCAGAAGGCAGTCATAGAGTAAC
18 S	AGTTCCAGCACATTTTGCGAG	TCATCCTCCGTGAGTTCTCCA

LCAT, lecithin cholesterol acyltransferase; LPCAT, Lysophosphatidylcholine Acyltransferase .

Table S2. The levels of TC, TG, HDL-C, LDL-C, ALT, and AST in serum.

Group	TC(mmol/L)	TG(mmol/L)	HDL-C(mmol/L)	LDL-C(mmol/L)	ALT(U/L)	AST(U/L)
Control	1.332±0.153	0.561±0.038	3.923±0.349	3.618±0.589	4.281±0.270	9.889±1.138
Model	2.842±0.311##	0.938±0.058##	2.990±0.227#	6.654±0.644##	4.576±0.319##	10.470±1.028
Fenofibrate	2.154±0.232*	0.728±0.068**	3.605±0.284*	4.845±0.617*	6.342±0.713	10.889±1.926
TUDCA-L	2.786±0.337	0.853±0.058	3.124±0.157	5.726±0.242	4.632±0.405	10.017±1.074
TUDCA-M	2.612±0.280	0.792±0.060*	3.380±0.427	5.600±0.386	4.725±0.334	10.303±1.376
TUDCA-H	2.226±0.207*	0.742±0.068**	3.559±0.232*	4.971±0.242*	4.473±0.267	10.421±0.976

Table S3. Detection of hyperlipidemia related metabolites in serum by UPLC-MS. Trend 1 is Control group compared with Model group; Trend 2 is Model group compared with TUDCA-H group.

No.	Metabolite name	TR (min)	m/z	Formula	VIP	*p* Value	Fold change	Trend 1	Trend 2	Scan mode
1	Sodium glycocholate	9.52	488.30	$C_{26}H_{43}NO_6$	2.93	1.18×10^{-3}	1.94	↓	-	+
2	PI(18:0/22:4(7Z,10Z,13Z,16Z))	15.36	937.57	$C_{49}H_{87}O_{13}P$	3.55	2.80×10^{-4}	3.95	↓	-	+
3	PI(16:1(9Z)/16:0)	12.97	853.51	$C_{41}H_{77}O_{13}P$	3.20	6.26×10^{-4}	3.44	↑	-	-
4	PE(18:1(9Z)/18:1(9Z))	13.70	744.55	$C_{41}H_{78}NO_8P$	1.37	4.22×10^{-2}	1.89	↑	-	+
5	PC(18:0/14:0)	13.17	756.55	$C_{40}H_{80}NO_8P$	1.44	3.64×10^{-2}	2.32	↑	-	+
6	Prostaglandin D1	7.94	353.23	$C_{20}H_{34}O_5$	3.31	4.89×10^{-4}	3.37	↑	-	-
7	Acetaminophen	17.33	210.06	$C_8H_9NO_2$	2.83	1.48×10^{-3}	1.05	-	↑	-
8	Threonic acid	0.86	137.04	$C_4H_8O_5$	3.90	1.25×10^{-4}	3.85	↓	-	+
9	3-Hydroxyisovaleric acid	14.74	117.06	$C_5H_{10}O_3$	3.45	3.51×10^{-4}	1.23	↑	↓	-
10	Hydroxykynurenine	0.49	223.07	$C_{10}H_{12}N_2O_4$	9.00	1.01×10^{-9}	1.53	-	↑	-
11	Estrone	3.99	293.15	$C_{18}H_{22}O_2$	2.34	4.53×10^{-3}	2.27	↑	-	+
12	Glycocholic acid	9.09	488.30	$C_{26}H_{43}NO_6$	6.00	1.01×10^{-6}	1.86	↓	-	+
13	Capric acid	0.76	217.1461	$C_{10}H_{20}O_2$	1.67	2.12×10^{-2}	1.42	-	↑	-
14	Acetaminophen glucuronide	3.12	326.0892	$C_{14}H_{17}NO_8$	1.7	2.01×10^{-2}	1.15	↓	-	-
15	Norcotinine	3.15	161.0721	$C_9H_{10}N_2O$	3.07	8.47×10^{-4}	1.43	-	↑	-

Table S4. Detection of hyperlipidemia related lipid metabolism in serum by UPLC-MS. Trend 1 is Control group compared with Model group; Trend 2 is Model group compared with TUDCA -H group.

No.	Metabolite name	TR (min)	m/z	Formula	VIP	*p* Value	Fold change	Trend 1	Trend 2	Scan mode
1	LysoPE(18:0/0:0)	4.02	481.3210	$C_{23}H_{48}NO_7P$	1.50	3.15×10^{-2}	2.55	-	↓	+
2	PE(16:1(9Z)/22:4(7Z,10Z,13Z,16Z))	2.89	765.5312	$C_{43}H_{76}NO_8P$	4.40	4.01×10^{-5}	9.06	-	↑	+
3	Pregnenolone sulfate	1.31	396.1972	$C_{21}H_{32}O_5S$	1.46	3.50×10^{-2}	1.26	-	↓	+
4	Glyceryl lactooleate	3.58	428.3153	$C_{24}H_{44}O_6$	2.65	2.22×10^{-3}	2.50	-	↑	+
5	LysoPE(22:6(4Z,7Z,10Z,13Z,16Z,19Z)/0:0)	4.22	525.2856	$C_{27}H_{44}NO_7P$	1.37	4.26×10^{-2}	1.40	-	↑	-
6	LysoPC(16:0/0:0)	2.99	495.3367	$C_{24}H_{50}NO_7P$	1.54	2.85×10^{-2}	0.88	-	↓	+
7	LysoPC(20:0/0:0)	4.96	551.4001	$C_{28}H_{58}NO_7P$	10.43	3.75×10^{-11}	1.74	-	↓	+
8	PC(18:3(9Z,12Z,15Z)/18:2(9Z,12Z))	7.04	779.5492	$C_{44}H_{78}NO_8P$	1.56	2.74×10^{-2}	0.71	↓	-	-
9	PC(16:0/16:0)	7.40	733.5686	$C_{40}H_{80}NO_8P$	2.01	9.84×10^{-3}	0.85	↑	-	+
10	LysoPC(0:0/18:0)	3.87	523.3686	$C_{26}H_{54}NO_7P$	2.50	3.13×10^{-3}	1.28	↓	-	+
11	Hypoxanthine	0.63	136.0396	$C_5H_4N_4O$	2.60	2.51×10^{-3}	0.23	↓	-	-
12	PC(16:0/18:0)	8.02	761.6001	$C_{42}H_{84}NO_8P$	5.44	3.64×10^{-6}	1.58	↑	-	+
13	SM(d18:1/14:0)	6.24	674.5401	$C_{37}H_{75}N_2O_6P$	10.24	5.75×10^{-11}	2.39	↑	-	+
14	SM(d18:1/22:0)	8.76	786.6684	$C_{45}H_{91}N_2O_6P$	12.41	3.93×10^{-13}	2.30	↑	-	+

Table S5. Detection of hyperlipidemia related metabolism in liver by UPLC-MS. Trend 1 is Control group compared with Model group; Trend 2 is Model group compared with TUDCA -H group.

No.	Metabolite name	TR (min)	m/z	Formula	VIP	*p* Value	Fold change	Trend 1	Trend 2	Scan mode
1	LysoPC(14:0/0:0)	9.11	467.2984	$C_{22}H_{46}NO_7P$	8.39	4.06×10^{-9}	1.99	↑	-	+
2	Stachydrine	0.70	143.0952	$C_7H_{13}NO_2$	8.33	4.72×10^{-9}	0.29	↓	-	+
3	Adipic acid	3.81	146.0572	$C_6H_{10}O_4$	6.11	7.75×10^{-7}	1.93	↑	↑	-
4	Taurolithocholic acid sulfate	10.55	563.2574	$C_{26}H_{45}NO_8S_2$	1.53	2.92×10^{-2}	0.82	↓	-	+
5	PC(18:1(11Z)/18:2(9Z,12Z))	13.73	783.5728	$C_{44}H_{82}NO_8P$	1.37	4.22×10^{-2}	2.03	↑	-	+
6	Arachidonic acid	13.03	304.2406	$C_{20}H_{32}O_2$	13.05	8.94×10^{-14}	3.84	-	↑	-
7	Creatine	0.66	131.0698	$C_4H_9N_3O_2$	1.63	2.33×10^{-2}	0.51	-	↓	+
8	Indolelactic acid	5.26	205.0735	$C_{11}H_{11}NO_3$	2.73	1.86×10^{-3}	2.01	-	↑	-
9	Adenosine	2.11	267.0981	$C_{10}H_{13}N_5O_4$	3.32	4.73×10^{-4}	0.64	-	↓	+
10	Cholesterol sulfate	12.69	466.3120	$C_{27}H_{46}O_4S$	1.77	1.68×10^{-2}	8.46	-	↑	-
11	Glycylleucine	3.48	188.1155	$C_8H_{16}N_2O_3$	6.32	4.80×10^{-7}	3.68	-	↑	-
12	D-Tryptophan	3.90	204.0895	$C_{11}H_{12}N_2O_2$	12.50	3.16×10^{-13}	4.11	-	↑	-
13	Glycodeoxycholic acid	7.16	449.3152	$C_{26}H_{43}NO_5$	6.26	5.48×10^{-7}	26.72	-	↑	-
14	4-Pyridoxic acid	1.88	183.0523	$C_8H_9NO_4$	4.48	3.35×10^{-5}	0.65	-	↓	-
15	Tauroursodeoxycholic acid	6.05	499.2969	$C_{26}H_{45}NO_6S$	2.97	1.07×10^{-3}	11.47		↑	-

Table S6. Detection of hyperlipidemia related lipid metabolism in liver by UPLC-MS. Trend 1 is Control group compared with Model group; Trend 2 is Model group compared with TUDCA -H group.

No.	Metabolite name	TR (min)	m/z	Formula	VIP	*p* Value	Fold change	Trend 1	Trend 2	Scan mode
1	PC(16:0/18:0)	7.88	807.5949	$C_{42}H_{84}NO_8P$	2.21	6.14×10^{-3}	1.34	↑	↓	-
2	PE(16:0/18:1(9Z))	7.76	717.5387	$C_{39}H_{76}NO_8P$	1.30	5.00×10^{-2}	0.98	-	↓	+
3	PC(16:0/16:0)	7.51	733.5705	$C_{40}H_{80}NO_8P$	1.69	2.04×10^{-2}	0.73	↑	-	+
4	PE(16:0/18:3(6Z,9Z,12Z))	6.98	713.5071	$C_{39}H_{72}NO_8P$	1.33	4.72×10^{-2}	1.41	-	↓	+
5	PE(18:3(6Z,9Z,12Z)/22:4(7Z,10Z, 13Z,16Z))	6.89	789.5282	$C_{45}H_{76}NO_8P$	3.21	6.13×10^{-4}	1.58	↑	-	-
6	PG(18:1(9Z)/18:1(9Z))	6.57	774.5371	$C_{42}H_{79}O_{10}P$	9.50	3.17×10^{-10}	2.57	↑	-	-
7	LysoPC(20:0/0:0)	5.17	551.4012	$C_{28}H_{58}NO_7P$	2.80	1.58×10^{-3}	2.07	↑	-	+
8	LysoPE(18:0/0:0)	4.44	481.322	$C_{23}H_{48}NO_7P$	2.95	1.13×10^{-3}	2.64	↑	-	+
9	LysoPC(0:0/18:0)	4.30	523.3694	$C_{26}H_{54}NO_7P$	2.36	4.37×10^{-3}	1.77	↑	-	+
10	Glyceryl lactooleate	3.81	428.3158	$C_{24}H_{44}O_6$	1.73	1.88×10^{-2}	0.42	↓	-	+
11	LysoPE(16:0/0:0)	3.34	453.2904	$C_{21}H_{44}NO_7P$	1.57	2.71×10^{-2}	1.21	↑	↓	+
